# The Quest for Comparability: Studying the Invariance of the Teachers’ Sense of Self-Efficacy (TSES) Measure across Countries

**DOI:** 10.1371/journal.pone.0150829

**Published:** 2016-03-09

**Authors:** Ronny Scherer, Malte Jansen, Trude Nilsen, Shaljan Areepattamannil, Herbert W. Marsh

**Affiliations:** 1 University of Oslo, Faculty of Educational Sciences, Centre for Educational Measurement at the University of Oslo (CEMO), Oslo, Norway; 2 German Institute for International Educational Research (DIPF), Berlin, Germany; 3 University of Oslo, Faculty of Educational Sciences, Department of Teacher Education and School Research, Oslo, Norway; 4 Emirates College for Advanced Education, Abu Dhabi, United Arab Emirates; 5 Australian Catholic University, Faculty of Health Sciences, Institute for Positive Psychology & Education, Sydney, Australia; 6 King Saud University, Riad, Saudi Arabia; 7 Oxford University, Department of Education, Oxford, United Kingdom; University of Rome, ITALY

## Abstract

Teachers’ self-efficacy is an important motivational construct that is positively related to a variety of outcomes for both the teachers and their students. This study addresses challenges associated with the commonly used ‘Teachers’ Sense of Self-Efficacy (TSES)’ measure across countries and provides a synergism between substantive research on teachers’ self-efficacy and the novel methodological approach of exploratory structural equation modeling (ESEM). These challenges include adequately representing the conceptual overlap between the facets of self-efficacy in a measurement model (cross-loadings) and comparing means and factor structures across countries (measurement invariance). On the basis of the OECD Teaching and Learning International Survey (TALIS) 2013 data set comprising 32 countries (*N* = 164,687), we investigate the effects of cross-loadings in the TSES measurement model on the results of measurement invariance testing and the estimation of relations to external constructs (i.e., working experience, job satisfaction). To further test the robustness of our results, we replicate the 32-countries analyses for three selected sub-groups of countries (i.e., Nordic, East and South-East Asian, and Anglo-Saxon country clusters). For each of the TALIS 2013 participating countries, we found that the factor structure of the self-efficacy measure is better represented by ESEM than by confirmatory factor analysis (CFA) models that do not allow for cross-loadings. For both ESEM and CFA, only metric invariance could be achieved. Nevertheless, invariance levels beyond metric invariance are better achieved with ESEM within selected country clusters. Moreover, the existence of cross-loadings did not affect the relations between the dimensions of teachers’ self-efficacy and external constructs. Overall, this study shows that a conceptual overlap between the facets of self-efficacy exists and can be well-represented by ESEM. We further argue for the cross-cultural generalizability of the corresponding measurement model.

## Introduction

Teachers’ self-efficacy has been in the focus of educational psychologists for many years. Specifically in the fields of teacher education and teacher effectiveness, the construct is considered to be an important correlate of teachers’ well-being, job satisfaction, instructional behavior, and students’ educational outcomes [1–6]. There is a consensus on its multidimensional nature, assuming at least three related but distinct facets that correspond to different teaching practices and aspects of teaching quality: self-efficacy in classroom management, instruction, and student engagement [6–8]. In order to assess these three facets, Tschannen-Moran and Woolfolk Hoy established the ‘Teachers’ Sense of Self-Efficacy (TSES)’ scale [6], which formed the basis for a number of studies that were specifically concerned with comparing the measurement of teachers’ self-efficacy across countries and cultures [9–12]. However, such cross-national comparisons, even though very interesting, rely on one key assumption: that the construct can be measured invariantly across countries, meaning that the same measurement model applies [13]. If the invariance assumption is violated, inferences on differences in teachers’ self-efficacy across countries are compromised [14].

Most previous studies that attempted to establish measurement invariance of the TSES scale provided evidence that at least the numbers of factors and the item-factor links (i.e., factor loadings) are comparable across some countries and cultures [10–12, 15]. However, higher levels of invariance that enable researchers to compare the means of teachers’ self-efficacy have rarely been met [10–12, 15]. This finding, however, may have different explanations. For example, Vieluf and colleagues [15] pointed to the existence of country-specific response styles as a source of non-invariance which could be due to cultural, educational, and language-related differences. Another reason for this lack of comparability may lie in too strict assumptions on item-factor links that neglected potential overlaps between the factors of teachers’ self-efficacy [16]. Such overlaps are likely to occur in the measurement of self-efficacy because the three aspects of teaching (i.e., classroom management, instruction, and student engagement) are not strictly distinct [17, 18]. Hence, there is a need for measurement models that systematically account for this potential overlap on the one hand, and establish the required invariance levels on the other hand.

Against this background, the present study aims to test the hypothesis of potential construct overlaps as manifested by significant cross-loadings in the TSES measurement models with respect to the factor structure and its measurement invariance across 32 countries and selected country clusters. Moreover, the consequences of such overlaps primarily for invariance testing and the correlations to external constructs (i.e., teachers’ work experience and job satisfaction) are investigated. We make use of the representative large-scale dataset of the OECD Teaching and Learning International Survey (TALIS) 2013, an international survey that provides opportunities for teachers and school leaders to indicate their perceptions of for instance the school as a learning environment, appraisal and feedback, teaching practices, leadership, self-efficacy, and job satisfaction [19]. The present study proposes a synergism between substantive research on the measurement of teachers’ self-efficacy and recent methodological advances in multi-group latent variable modeling [20]. Specifically, we apply the relatively new approach of exploratory structural equation modeling to a substantive field that has received increasing attention in educational psychology and educational large-scale assessments, and demonstrate its flexibility in handling the structure and invariance of the TSES measure.

### Teachers’ Self-Efficacy

Currently, there is an enhanced awareness of the importance of teachers’ personality and beliefs, particularly in the fields of teacher education and effectiveness [6, 7]. There might be a number of reasons for this increased attention: First, teachers’ self-efficacy is regarded as an essential teacher characteristic which is related to their effective behavior in classroom settings [21]. Second, these teaching practices, in turn, affect students’ educational outcomes such as achievement and motivation [1, 22, 23]. Third, teachers with high self-efficacy show higher job satisfaction and are less likely to be affected by burnout [24, 25]. Fourth, teachers’ levels of self-efficacy may change with their work experience over time and may therefore indicate changes in their professional competences, job satisfaction, and well-being [26–28]. Consequently, the construct has received much attention in both national and international assessments [7, 9, 19]. For instance, besides investigating teachers’ characteristics, professional development, appraisal and feedback, and perceptions of school leadership, TALIS has put emphasis on the assessment of teachers’ self-efficacy and related constructs such as their job satisfaction as important outcome variables [19]. This emphasis is not surprising, given that teachers’ self-efficacy relates to their instructional practices and student achievement [2, 3].

On the basis of social cognitive theory, Bandura [29] defined self-efficacy beliefs as individuals’ perceptions of their capabilities to plan and execute specific behavior. These perceptions can therefore be regarded as personal beliefs about what that person *can do* rather than beliefs about what he or she *will do* [30]. In consequence, self-efficacy beliefs affect a person’s goals, actions, and effort [25]. Bandura [29] further pointed out that these beliefs are not merely perceptions of external factors and obstacles that might facilitate or inhibit the execution of behaviors, but should be regarded as self-referent; they are first and foremost subjective evaluations of one’s own capability, although they are formed and affected by external factors [11, 31]. Put differently, people that are subject to the same environment (e.g., a school or country) may show very different efficacy beliefs. Moreover, environments may also affect collective efficacy beliefs leading to systematic differences between groups (e.g., teachers in different countries). Following Bandura’s definition, teachers’ self-efficacy is conceptualized as their beliefs in their capabilities to enact certain teaching behavior that may influence students’ educational outcomes, such as achievement, interest, and motivation [5–7]. Tschannen-Moran and Woolfolk Hoy [32] as well as Malinen and colleagues [33] emphasized that these beliefs are context-specific and connected to instructional capabilities and tasks. Consequently, different beliefs may result from different teaching environments and practices [7]. Existing research has therefore aligned the conceptualization and measurement of teachers’ self-efficacy with specific teaching practices and requirements to enhance student learning [1, 3, 10, 34, 35]. In this sense, the conceptualization of the construct consequently comprises elements of self-efficacy theory, and is also informed by research on teaching quality in which specific criteria for effective instruction are defined and operationalized [36].

#### A Conceptual Framework of Teachers’ Self-Efficacy

In a number of studies, researchers have described teaching quality as a concept that comprises different teaching practices and aspects of instruction. For instance, high quality classrooms provide an orderly learning environment, devoid of disruptive behavior, and contain cognitively activating tasks as well as opportunities in which students are engaged and motivated to learn [37]. Although there have been a number of conceptualizations of teaching quality, describing different aspects of teaching, its multidimensionality can be regarded as a common characteristic [18, 36, 38, 39]. In a parallel line of research with a view to aligning teaching practices with self-efficacy beliefs, Tschannen-Moran and Woolfolk Hoy [6] proposed a multidimensional framework of teachers’ self-efficacy; Skaalvik and Skaalvik [25] strengthened this approach and argued that considering the construct to be unidimensional was a major limitation in self-efficacy research. Hence, there have been concerted efforts to differentiate between at least the three pertinent factors of teachers’ self-efficacy that Tschannen-Moran and Woolfolk Hoy [6] identified: Self-efficacy in classroom management, instruction, and student engagement [7, 8, 11, 12, 25, 33]. *Teachers’ self-efficacy in classroom management* refers to their capabilities for establishing an orderly environment without disruptions and coping with disruptive behavior [40]; *self-efficacy in instruction* refers to a broad understanding of instruction which focuses on the use of alternative teaching practices, assessment strategies, and explanations; *self-efficacy in student engagement* addresses emotional and cognitive support for students and includes capabilities to motivate students for learning. Given the multidimensional nature of teachers’ self-efficacy, it is important to account for these interrelated yet disparate factors in the measurement of the construct [6].

#### The Teachers’ Sense of Self-Efficacy Measure

In congruence with the multidimensional approach to measuring teachers’ self-efficacy, Tschannen-Moran and Woolfolk Hoy [6] developed the *Teachers’ Sense of Self-Efficacy (TSES)* scale that measures the three aforementioned factors pertaining to teachers’ self-efficacy. This scale is commonly used in self-efficacy research and has been validated among a number of teacher samples with respect to the existence of three correlated factors and the relations to constructs such as job satisfaction and work experience [10–12]. However, an aspect that has been neglected while validating this scale relates to construct overlaps. In particular, given that teachers’ practices of classroom management, instruction, and student engagement may go together and are conceptually related, items measuring their self-beliefs may not be exclusively related to one factor, but also include aspects of the other two [27]. For instance, a closer examination of the items, ‘*I can craft good questions for my students*’ and ‘*I can help students think critically*’ [12, 19], which were assigned to ‘*Self-Efficacy in Instruction*’ and ‘*Self-Efficacy in Student Engagement*’ respectively, suggests that they may not refer to only one factor, because teaching practices for enhancing critical thinking may go together with practices of crafting good questions to cognitively activate students’ learning processes [41, 42]. From a methodological point of view, the existence of such an overlap between the TSES factors should manifest not only in high factor correlations but also in an improvement in goodness-of-fit, especially when employing models that allow items to belong to more than one of the three factors [43]. Until now, confirmatory factor analysis (CFA) has been used to model the structure of the teachers’ sense of self-efficacy measure under the assumption that the item-factor links are perfect and overlaps do not exist. In response to this practice, Duffin and colleagues [27] suggested validating the structure of the TSES measure by exploratory factor-analytic approaches to uncover item cross-loadings. Following the same line of argumentation, Marsh et al. [44] argued that the assumption of perfect item-factor links might be too strict for some psychological constructs, and therefore recommended using approaches such as exploratory structural equation modeling. Although test developers may suggest excluding items belonging to more than one factor, allowing overlaps might be reasonable to represent the conceptual breadth of the construct [16]. For example, only using items related to emotional rather than cognitive student engagement might lead to less cross-factors relations to self-efficacy in instruction; however, it would also compromise an important aspect of student engagement that is part of the construct definition [45]. The degree to which such an overlap between the factors of self-efficacy exists empirically, and how it affects the measurement of the construct has not yet been fully explored.

### Cross-National Perspectives of Teachers’ Self-efficacy

In psychological and social science research–and public policy more generally–there is a pre-occupation with cross-cultural differences rather than of cross-cultural generalizability. Fueled in part by a null hypothesis testing perspective, given a sufficiently large sample size there will almost always be statistically significant cross-cultural differences for most variables–even if the effect is so trivially small as to have no substantive implications. It is very difficult to prove the null hypothesis. However, this focus on cross-cultural differences tends to ignore the strong support for cross-cultural similarities and the sometimes small sizes of cross-cultural differences.

Cross-cultural comparisons provide researchers a valuable, heuristic basis to test the external validity and generalizability of their measures, theories, and models. Matsumoto ([46], pp. 107–108] argued that: “Cultural differences challenge mainstream theoretical notions about the nature of people and force us to rethink basic theories of personality, perception, cognition, emotion, development, social psychology, and the like in fundamental and profound ways.” In their influential overview of cross-cultural research, Segall, Lonner, and Berry ([47], p. 1102) stated that cross-cultural research’s three complementary goals were: “to transport and test our current psychological knowledge and perspectives by using them in other cultures; to explore and discover new aspects of the phenomenon being studied in local cultural terms; and to integrate what has been learned from these first two approaches in order to generate more nearly universal psychology, one that has pan-human validity.” Similarly, Sue [48] argued that researchers have not taken sufficient advantage of cross-cultural comparisons that allow researchers to test the external validity of their interpretations and to gain insights about the applicability of their theories and models.

Cross-national perspectives on teachers’ self-efficacy are therefore considered valuable to study how ability beliefs generalize across countries, cultures, and educational settings on the one hand, and the validity of the measurement in terms of its comparability on the other hand [11]. Because there are considerable variations in teaching practices and conditions that may affect and change teachers’ ability beliefs, researchers tend to place emphasis on the importance of incorporating cross-national perspectives into self-efficacy research (see [10]). A number of studies have therefore compared different countries from Western, Asian, and other cultures [10–12, 15, 33, 49]. Among others, Oettingen [50] and Vieluf et al. [15] provided potential explanations for the differences that were found in these studies. They pointed out that those differences may be culturally-driven, and may tap into the following dimensions: collectivism/individualism, power distance, uncertainty avoidance, and masculinity/femininity. From an educational perspective on self-efficacy, differences may also occur due to differences in professional teacher education, teaching practices, school conditions, or educational beliefs [24, 51–53]. Vieluf and colleagues [15] added further dimensions and argued that cultural differences in teachers’ self-efficacy might interact with differences in value orientations and specific tendencies of perceptions of oneself which are oriented towards culture-specific standards. Such differences may manifest in differences in response styles [13]. Against this background, it appears reasonable to assume cross-national differences in teachers’ self-efficacy.

Furthermore, there is growing evidence that the multidimensional nature of teachers’ self-efficacy persists across countries and cultures [10, 12, 33]. Klassen and colleagues [11] studied diverse countries with respect to the structure of and performance on the TSES measure. Although these cultures differed considerably in their teaching practices and cultural beliefs, the structure of the self-efficacy measure remained robust, lending evidence on the generalizability of the measurement. Nonetheless, they also observed that response tendencies differed, thus compromising full comparability of the measurement if single items are affected by these tendencies. This finding supports Vieluf and her colleagues’ [15] observations of different response styles in a unidimensional measure of self-efficacy that explained the lack of multilevel isomorphism in their study. From a measurement perspective, it may therefore not be advisable to compare the means of the three TSES factors across countries and cultures because these three factors may have different meanings across countries and cultures [13, 14]. More recently, Desa [9] provided support for this claim by demonstrating that the invariance levels necessary to conduct mean comparisons were rarely met for perception-based measures of teacher characteristics. Hence, non-invariance can be regarded as a serious challenge in comparative studies on teachers’ self-efficacy.

While attempting to align the measurement of teachers’ self-efficacy along the lines of cross-national perspectives on teacher self-efficacy beliefs, it is still unclear to what extent the assumption of perfect item-factor links could undermine or improve the invariance and generalizability of the TSES measure.

### Exploratory Structural Equation Modeling (ESEM)

**Fig 1:**
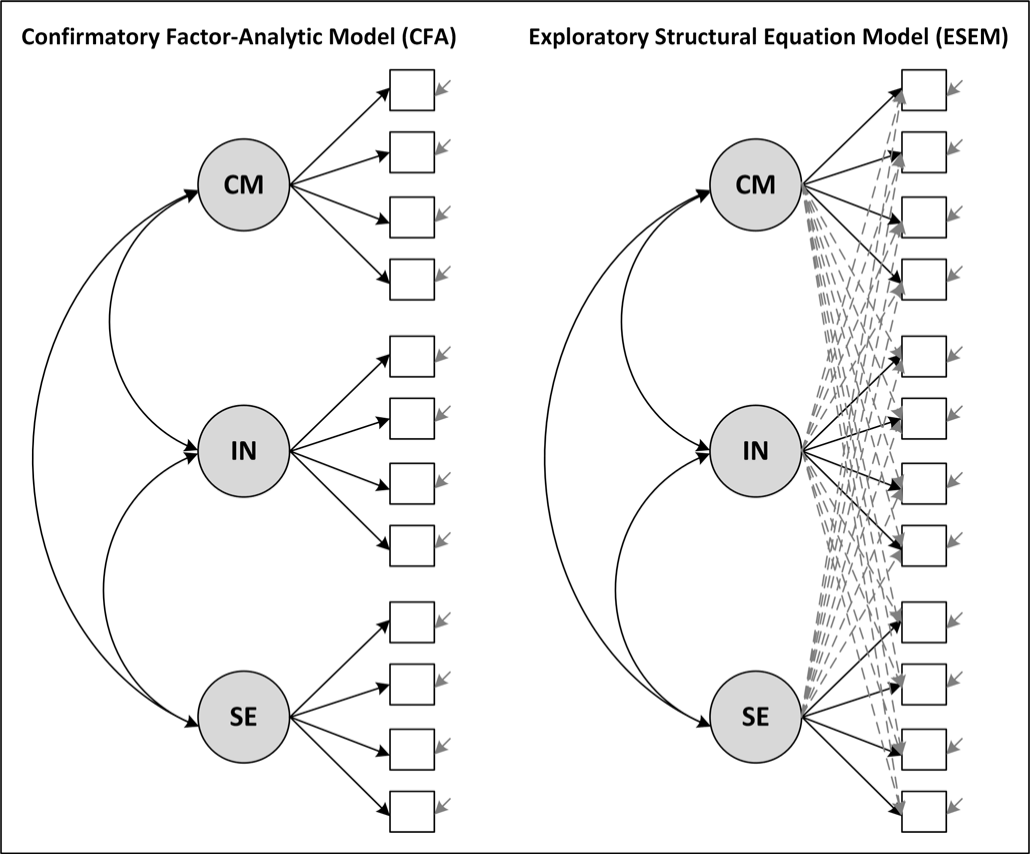
Measurement Models of the CFA and ESEM Approaches. *Note*. CM = Self-efficacy in classroom management, IN = Self-efficacy in instruction, SE = Self-efficacy in student engagement. Dashed lines indicate cross-loadings.

As mentioned earlier, confirmatory factor analysis (CFA) is traditionally used to test specific hypotheses on the factor structure of construct measures and measurement invariance [44]. However, this approach assumes a simple structure of the data, that is, a unique link between items and latent variables (i.e., factors) without any cross-loadings (Fig 1). Regarding the construct overlap, one would expect models, which allow for cross-loadings, to represent the structure of the TSES measure more appropriately than CFA models without cross-loadings [16].

Marsh and colleagues [43] proposed an approach, which combines the features of exploratory and confirmatory factor analysis but is still flexible enough to be extended to structural equation models. This exploratory structural equation modeling (ESEM) approach was studied in different substantive areas and allows researchers to introduce covariates, correlated residuals, and to test for different levels of measurement invariance across groups [44]. Research has indicated that factor correlations and relations to external variables are not overestimated in the ESEM approach [54].

Technically speaking, ESEM freely estimates all rotated cross-loadings that occur between items and factors (see Fig 1). In the first step, the unconstrained factor structure is estimated. This preliminary structure is rotated in the second step by using a wide range of methods such as oblique or orthogonal rotations (for details, please refer to [54]). For instance, the oblique target rotation method assumes cross-loadings, which can be specified as being approximately zero. In the final model, however, these target loadings may result in values that significantly deviate from zero [54]. The target rotation allows researchers to incorporate a-priori assumptions on the factor structure, and can be regarded as an approximation of confirmatory factor analysis with exact zero cross-loadings [44]. A more detailed description of ESEM and the rotation methods can be found in Marsh et al. [44]. When testing for different levels of measurement invariance, the rotation in ESEM is employed in such a way that not only the main but also the cross-loadings are constrained across groups. Hence, to investigate measurement invariance of teachers’ self-efficacy while taking into account the overlap between factors at the same time, ESEM provides an appropriate analytical approach.

### The Present Study

We identified two challenges regarding the measurement of teachers’ self-efficacy with the TSES scale: First, the facets of self-efficacy might not be strictly distinct due to their conceptual overlap. Methodologically speaking, the link between items and factors may not be perfect [24] and the assumption of an absence of cross-loadings that is inherent in traditional CFA models may be violated. Second, scalar and strict invariance of ratings on instructional abilities across countries have rarely been achieved, which has compromised mean comparisons of teachers’ self-efficacy across countries [10–12, 15]. However, being able to conduct such comparisons would shed light on differences between educational systems with respect to teachers’ self-beliefs [7, 10, 15, 55]. The present investigation is therefore aimed at addressing these challenges by comparing the ESEM approach with the CFA approach with respect to their appropriateness in describing the factor structure and cross-country measurement invariance of the widely used TSES scale.

#### Research questions

We compare the factor structure of the teachers’ sense self-efficacy measure across the 32 participating TALIS 2013 countries on the one hand and three selected country clusters on the other hand by using both the ESEM and the CFA approach. These are the country clusters we refer to: Nordic cluster (Denmark, Finland, Norway, and Sweden), East and South-East Asian cluster (Japan, Korea, Malaysia, and Singapore), and Anglo-Saxon cluster (Australia, England, and the United States of America) clusters. Specifically, our aim with transferring the proposed modeling approaches to these selected country clusters was to validate the findings obtained from examining the total sample. For a more elaborate discussion on this choice, please refer to the section below. Finally, we evaluated the consequences of existing cross-loadings for the correlations to external constructs. In particular, we address the following research questions:

- *To what extent does exploratory structural equation modelling*, *which allows for item cross-loadings*, *represent the factor structure of the TSES measure more appropriately than confirmatory factor analysis*? *(Research Question 1)*
- *Which levels of measurement invariance of the TSES measure can be achieved across countries for both the CFA and ESEM approach*? *(Research Question 2)*
- *If at least metric invariance can be established*, *to what extent do ESEM and CFA differ in the estimation of the correlations between the TSES factors*, *teachers’ years of work experience*, *and their job satisfaction*? *(Research Question 3)*

#### Selection of country clusters

Generally speaking, we argue that examining the findings obtained from the total TALIS 2013 sample for selected country clusters addresses the generalizability of the results [56, 57]. Specifically, by answering our research questions for sub-samples that were selected for substantive reasons (see below), we may also provide some evidence on the robustness of our results. Nevertheless, as the present study does not focus on the substantive interpretation of country differences but the comparison of different modeling approaches, an in-depth discussion of such differences is beyond the scope of this paper.

In order to enable a comparison between countries of similar cultures, languages, and educational contexts, and yet at the same time to retain a global perspective, we chose to select three country clusters on the basis of substantive theory. First, according to Bulle’s [58] review of OECD ideal-typical educational models, countries can be classified with respect to their dominant educational structures and objectives. Specifically, distinguishing between educational objectives in a country that are centered on academic educational programs or on students’ general competencies as educational outcomes, Bulle presents a typology that identifies a number of country clusters. Among others, she specifies the Northern model (e.g., Denmark, Finland, Iceland, Norway, and Sweden), the Anglo-Saxon model (e.g., Australia, Canada, Ireland, New Zealand, United Kingdom, and the United States), and the East-Asian model (e.g., Japan and Korea). As the TALIS 2013 results indicated that teachers’ self-efficacy is related to school-related but also institutional factors [19], we argue that similarities in educational systems may result in similarities in the levels of self-efficacy (see also [59]). In fact, for at least the Anglo-Saxon and Nordic countries participating in TALIS 2013, similarities in the performance on single TSES items could be identified (for details, please refer to [19], Annex C); moreover, for the TALIS 2008 participating countries, a strong Nordic cluster was apparent [15].

Second, in light of existing research on cross-cultural differences in teachers’ self-efficacy, teachers from different clusters may understand the self-efficacy items differently [11], possibly as a result of different cultural differences [10, 15]. Thus, meaningful mean comparisons across clusters may hardly be possible, as this becomes an issue beyond statistical invariance. We consequently argue that cross-country comparisons *within* the three clusters, thereby controlling for the effects of major language, cultural and educational differences, might be more meaningful than *between* the clusters. However, at least to some extent, we expect teachers’ self-efficacy to show some variation even within the clusters. For instance, although the Nordic countries have similar languages, educational systems, and cultures from an international perspective [60], students’ performance and motivation in mathematics, teachers’ education and professional development differ [61]. These differences may lead to differences in teachers’ perceptions about their capabilities in teaching.

Third, the selected country clusters were by and large in the main focus of existing research on the factor structure of the TSES measure (e.g., [10–12, 33]), thus providing the basis for comparing the results obtained from these studies with those obtained from the present study.

## Materials and Method

### Sample and Procedure

Following our outline, we used the entire TALIS 2013 sample comprising the data from 32 countries and sub-national entities (*N* = 170,020) in order to address our research questions. The country data sets were based on the data obtained from nationally representative samples; the OECD has released these data sets as public-use files (available at http://stats.oecd.org/Index.aspx?datasetcode=talis_2013, accessed: 8/9/2015). The participating teachers worked in schools that provided ISCED level 2 education (rural and public schools; for details, please refer to [62], chap 5) and took questionnaires, which included, among others, questions on their self-efficacy and background. Of the total sample, 5,333 teachers did not respond to at least one item of the self-efficacy scale and were therefore excluded from the analyses, resulting in a total sample size of 164,687 teachers. The sample used in the present study included teachers from all subject areas in 1,808 schools [19]. Descriptive statistics and the resulting sample sizes are reported in Table 1. Test administration, coding of responses, and data preparation were employed according to the pre-defined TALIS 2013 quality standards [62]. Prior to the main survey, the “TALIS Board of Participating Countries (BPC)” which was commissioned by the OECD approved the standards concerning survey ethics, confidentiality, and survey operations for the study [62]. Since the current study conducts secondary data analyses of the publicly available TALIS 2013 data files, which were released to address research questions beyond the ones covered by the OECD report, it relies on the approval of research ethics by the BPC (see S3 Table).

**Table 1:** Descriptive Sample Statistics and Scale Reliabilities.

*Country*	*N*	*Females [%]*	*Age [years] M (SD)*	*Scale Reliability* ω		
				*Classroom Management*	*Instruction*	*Student Engagement*
Australia ^a^	6,271	57	43.2 (11.5)	.87	.83	.87
Brazil	13,334	68	39.5 (9.5)	.84	.83	.84
Bulgaria	2,953	82	47.6 (9.1)	.82	.81	.84
Chile	1,543	62	41.3 (11.9)	.88	.84	.81
Croatia	3,626	74	42.6 (11.7)	.87	.80	.78
Czech Republic	3,204	75	43.8 (10.8)	.87	.77	.82
Denmark ^b^	5,051	62	45.7 (10.6)	.88	.76	.82
Estonia	3,057	83	47.9 (11.2)	.81	.78	.77
Finland ^b^	11,097	72	44.4 (10.1)	.89	.81	.85
France	2,808	66	42.1 (9.8)	.85	.69	.81
Israel	3,229	75	41.9 (10.3)	.89	.83	.85
Italy	6,846	72	48.9 (8.8)	.84	.80	.84
Japan ^c^	3,463	40	42.0 (10.9)	.90	.87	.80
Korea ^c^	2,825	70	42.5 (9.1)	.91	.87	.84
Latvia	4,173	88	47.4 (10.1)	.81	.75	.78
Malaysia ^c^	2,953	71	39.0 (8.5)	.89	.89	.87
Mexico	9,465	52	42.1 (10.4)	.84	.83	.76
Netherlands	1,788	54	43.3 (11.9)	.90	.62	.78
Norway ^b^	7,501	64	45.4 (11.3)	.86	.76	.81
Poland	10,189	76	42.5 (9.0)	.84	.81	.80
Portugal	6,704	72	45.0 (7.6)	.88	.84	.84
Serbia	3,819	66	43.0 (10.8)	.82	.79	.83
Singapore ^c^	10,302	64	36.7 (9.8)	.89	.86	.89
Slovak Republic	3,454	81	43.5 (10.9)	.84	.82	.81
Spain	9,261	59	45.6 (8.6)	.87	.81	.83
Sweden ^b^	3,160	66	45.9 (10.5)	.88	.78	.80
United States of America ^a^	1,854	66	42.2 (11.3)	.86	.82	.88
Sub-national entities						
England (United Kingdom) ^a^	2,348	64	39.3 (10.4)	.88	.81	.86
Flanders (Belgium)	5,671	74	39.2 (10.5)	.90	.74	.80
Abu Dhabi (United Arab Emirates)	4,530	55	39.7 (8.6)	.87	.84	.83
Alberta (Canada)	1,718	61	40.0 (10.2)	.88	.83	.86
Romania	6,490	70	43.0 (10.9)	.85	.82	.81
Total TALIS 2013 Sample	164,687	67	42.9 (10.5)	.85	.83	.85

*Note*. Scale reliabilities are reported as McDonald’s ω.

^a^ Anglo-Saxon country cluster

^b^ Nordic country cluster

^c^ East and South-East Asian country cluster.

The translation of the measures used in TALIS 2013 was closely monitored and specific standards had to be fulfilled by the translation services conducted in the participating countries. Moreover, psychometric methods were used to ensure that the translation of the instruments into different languages provided comparable measures. Please find more detailed information on the specifics of the item translation processes in the TALIS 2013 technical report [62].Measures

#### Teachers’ self-efficacy

On the basis of a short form of the ‘*Teachers’ Sense of Efficacy (TSES)*’ scale [6, 12] that distinguishes between teachers’ self-efficacy in classroom management, instruction, and student engagement, TALIS 2013 asked teachers to rate four statements for each of the three factors according to the extent to which they believed in their capabilities for doing the tasks (*1 = not at all*, *4 = a lot*). The item wordings are shown in the S1 Table. In this paper, we use the original item labels assigned by TALIS 2013 [62]. To evaluate the reliability of the three self-efficacy factors, we used McDonald’s ω for polychoric correlation matrices [63]. We found reasonable up to high reliabilities for each factor across countries (see Table 1).

#### Work experience

Teachers’ work experience was indicated by the number of years they have been working in the teaching profession until the administration of the TALIS 2013 questionnaire. For the total TALIS 2013 sample, teachers spent on average 16.2 years (*SD* = 10.3 years) in their profession.

#### Job satisfaction

As an external variable that has been heavily studied in order to validate the teachers’ sense of self-efficacy questionnaire, we included teachers’ job satisfaction in our analyses [1, 6, 25]. Specifically, job satisfaction was defined as “the sense of fulfilment and gratification from working in an occupation” ([19], p. 182]. In this study, we refer to teachers’ satisfaction with their profession and use their responses to six items were administered in order to assess job satisfaction with the teacher profession, three of which were formulated positively (e.g., “*The advantages of being a teacher clearly outweigh the disadvantages*”) and negatively (e.g., “*I regret that I decided to become a teacher*”). Teachers had to rate these statements on a four-point Likert scale (0 = *strongly disagree*, 3 = *strongly agree*). The resulting responses formed a scale that showed good reliabilities across countries (reported Cronbach’s α > .72 for all countries except for Mexico; see TALIS 2013 technical report; [62], pp. 206–216). Moreover, for the measurement of teachers’ job satisfaction, metric invariance could be established across the 32 participating TALIS 2013 countries [9].

### Statistical Analyses

#### Measurement models and estimator

We tested whether or not the theoretically implied, three-dimensional structure of the TSES measure held (Research Question 1) by specifying a confirmatory-factor analytic (CFA; no cross-loadings) and an exploratory structural equation model (ESEM; with cross-loadings) with three correlated factors (Fig 1). For the latter, we used the oblique target rotation, because we assumed correlated factors of self-efficacy with cross-loadings close to zero. This choice was basically made in light of our expectation that the self-efficacy items will mainly load on the factors they have originally been assigned to, but show lower loadings on the other factors. Moreover, Marsh et al. [44] argued that target rotation produces less bias in model parameters such as factor loadings than, for example, Geomin rotation. This particularly applies to factors with a small number of indicators.

In order to evaluate the goodness-of-fit for the CFA and the ESEM approach, we referred to common guidelines (i.e., CFI ≥ .95, TLI ≥ .95, RMSEA ≤ .08, SRMR ≤ .10 for an acceptable model fit; [64]). In all analyses, robust maximum likelihood estimation (MLR) with standard errors and tests of fit that were robust against non-normality of observations and the use of categorical variables in the presence of at least four response categories was used [65]. This choice was also driven by the fact that the MLR continuous estimation can handle missing values that are missing at random more appropriately than, for instance, the categorical weighted least squares means and variance adjusted (WLSMV) estimation [66].

#### Measurement invariance testing

We tested the measurement model obtained from the results on Research Question 1 for configural, metric, scalar, and strict invariance by systematically constraining factor loadings, item intercepts, and item uniquenesses (i.e., item-specific residual variances) to equality across countries [14] in order to address Research Question 2. Although different practices of invariance testing have been proposed, there are at least four levels of invariance [43]: The first refers to *configural invariance*; configural invariance is established when the same numbers of factors are present in each group and these factors are defined in the same way (i.e., the items are assumed to load on the same factors in all groups). In a configural invariance model, all model parameters (e.g., factor loadings, intercepts, factor variances) are freely estimated in each group. On the second level of invariance, factor loadings are constrained to equality, putting the latent factors on the same scale (*metric invariance*). This constraint is also applied in ESEM, resulting in the equality of all factor loadings including the cross-loadings. If metric invariance can be established, the factor correlations and relations to external constructs may be compared across groups [14]. In fact, in order to compare the relations among the three factors of teachers’ self-efficacy and their correlations with teachers’ work experience and job satisfaction across the TALIS 2013 countries, multi-group CFA and ESEM models, both assuming metric invariance across countries, will be specified. Third, besides equal factor loadings, item intercepts are constrained in the *scalar invariance* model. This model forms the prerequisite of comparing factor means across groups [14]. The fourth model of *strict invariance* constrains the item uniquenesses (i.e., residual variances), facilitating comparisons of manifest means [43]. Since this level is hardly achieved in studies comparing more than two culturally diverse countries [15, 67], Byrne, Shavelson, and Muthén [68] recommended relaxing the assumption of equal item intercepts by freeing some of the intercepts, suggesting a *partial scalar invariance* model. Given the number of countries in the total TALIS 2013 sample and the resulting number of possible combinations to free the item intercepts, we only tested for this type of invariance within the selected country clusters.

We evaluated the invariance models on the basis of their goodness-of-fit and the results of comparisons between the fit of adjacent models [43]. However, we did not consider χ^2^ difference testing for interpreting the fit of nested models, because the χ^2^ statistic strongly depends on the sample size [67]. As an alternative, we inspected the changes in incremental fit indices after adding parameter constraints to a model. Chen [69] suggested specific cut-off values that may indicate substantial deviations from the assumption of invariance. Specifically, in comparison to the less restrictive model, a decrease in the CFI and TLI of less or equal than .010, an increase in the RMSEA of less or equal than .015, and, finally, and increase in the SRMR of equal or less than .030 may be considered practically insignificant changes in model fit; thus allowing researchers to accept the invariance model with more restrictions on parameters. Although these guidelines have been widely applied in educational measurement, they have been validated mostly in two-group scenarios (see also [70]). Moreover, the performance of these cut-off values varies with respect to sample size, the number of factors in the measurement model, the treatment of the data (i.e., continuous vs. categorical treatment of teacher responses), the number of groups, the type of measurement invariance tested, and the factor structure specified [67, 69–71]. In fact, Khojasteh and Lo [71] showed that less restrictive cut-offs for the RMSEA and the SRMR should be applied in bifactor structures, which are comparable to factor models with cross-loadings (i.e., .034 and .030, respectively). Desa [9] argued even further and accepted changes in the CFI below .015 in large multi-group samples such as the one obtained from TALIS 2013. In light of the complexity of the sample (i.e., large-scale data, complex sampling procedure, more than 2 groups) and the factor models (in particular, ESEM) in the current study, we operationally apply the following cut-offs when comparing the more restrictive with the less restrictive invariance models: ΔCFI ≤ .010, ΔTLI ≤ .010, ΔRMSEA ≤ .015, and ΔSRMR ≤ .030. However, it is emphasized that these cut-off values constitute rough guidelines only, rather than “golden rules” [72]. Hence, small deviations from these values, yet in only one of the fit statistics (up to +.005), may still be accepted.

#### Sampling procedure, selection bias, hierarchical structure, and missing data

In TALIS 2013, teachers and schools were randomly selected in a two-step probability sampling design. Specifically, teachers (secondary sampling units) were randomly selected from a list of in-scope teachers for each school that has been randomly selected within a country (primary sampling units; [62], p. 73). In this context, an “in-scope teachers” was defined as “(…) a person whose professional activity involves the planning, organising and conducting of group activities whereby students’ knowledge, skills and attitudes develop as stipulated by educational programmes. In short, it is one whose main activity is teaching (…)” ([62], p. 74). Due to different selection probabilities and the sampling of schools and teachers, sampling errors may occur. We used teachers’ final weights in all analyses in order to correct for potential selection bias and balance differences in sample sizes [62, 73]. These weights comprise sub-weights that account for the different probabilities of being selected as a school and being selected as a teacher in a selected school within a country (for further details, please refer to [62]). Moreover, we accounted for the hierarchical data structure (i.e., teachers nested in schools) by adjusting the standard errors of all model parameters in the statistical package M*plus* 7.2 (TYPE = COMPLEX option; [74]). Moreover, the χ^2^ values for the models specified were corrected using the formula by Satorra and Bentler [75]. Given that we apply multi-group modeling approaches (i.e., multi-group CFA and ESEM) to investigate the invariance of the TSES measurement model across countries (Research Question 2) and the relations to external constructs (Research Question 3), the country level is taken into account as the level of grouping. In light of the relatively small number of countries participating in TALIS 2013, we treated country effects as fixed rather than random in the multi-group approach [76].

Among the teachers who took the questionnaire on self-efficacy, the percentage of missing values ranged between 1.9–2.1% for the items. These missing values were not due to the design of the study. Hence, we assumed that they occurred randomly and consequently applied full-information maximum likelihood estimation [77]. As the present investigation was undertaken with a large-scale data set and the specified models comprised a moderate number of parameters, we chose the 1% level of significance.

## Results

### Factor structure (Research Question 1)

To check whether or not the structure of the TSES measure with a perfect item-factor link was supported by the data (Research Question 1), we fitted a CFA model to the total sample in a first step and assumed three correlated traits, each representing one factor of self-efficacy. In this model, cross-loadings were fixed to zero (see Table 2, CFA). The resulting model fitted the data reasonably (see Table 2). In a second step, we applied ESEM and loosened the assumption of a perfect item-factor link by using an oblique target rotation. The model fitted the data well (see Table 2, ESEM) and significantly outperformed the CFA model, as indicated by the remarkable reduction of the χ^2^ value, the lower values of the RMSEA and SRMR, and higher values of the CFI and TLI, ΔCFI = +.036, ΔTLI = +.036, ΔRMSEA = –.008, ΔSRMR = –.027. With respect to cross-loadings, the ESEM approach revealed significant values up to .31 (see Table 2). According to the resulting factor loadings, items can be grouped into two categories: (a) Items with the highest loading on the originally assigned factor and very low cross-loadings (e.g., TT2G34H, TT2G34K); (b) Items with the highest loading on the originally assigned factor but substantial cross-loadings (e.g., TT2G34C, TT2G34G). The existence of items belonging to the second category indicates an overlap between the self-efficacy factors. This overlap was particularly apparent between the factors of ‘Instruction’ and ‘Classroom Management’ as well as ‘Instruction’ and ‘Student Engagement’, and consequently led to lower factor correlations in the ESEM model (see Table 2). For both the ESEM and CFA approach, the highest correlation was found between ‘Instruction’ and ‘Student Engagement’.

**Table 2:** Standardized Factor Loadings, Factor Correlations, and Fit Indices of the CFA and ESEM Approaches for the Total TALIS 2013 Sample.

	*CFA*			*ESEM*		
*Item*	*Factor 1*	*Factor 2*	*Factor 3*	*Factor 1*	*Factor 2*	*Factor 3*
*Factor 1*: *Classroom Management*						
TT2G34D	.75 (.01)*	–	–	.77 (.01)*	–.06 (.01)*	.03 (.01)*
TT2G34F	.65 (.01)*	–	–	.50 (.01)*	.16 (.01)*	.04 (.01)*
TT2G34H	.80 (.01)*	–	–	.83 (.01)*	–.04 (.01)*	.01 (.01)
TT2G34I	.76 (.01)*	–	–	.81 (.01)*	–.01 (.01)	–.05 (.01)*
*Factor 2*: *Instruction*						
TT2G34C	–	.65 (.01)*	–	.06 (.01)*	.34 (.01)*	.31 (.02)*
TT2G34J	–	.71 (.01)*	–	.07 (.01)*	.65 (.01)*	.00 (.01)
TT2G34K	–	.73 (.01)*	–	.04 (.01)*	.77 (.01)*	–.06 (.01)*
TT2G34L	–	.76 (.01)*	–	–.06 (.01)*	.77 (.01)*	.04 (.01)*
*Factor 3*: *Student Engagement*						
TT2G34A	–	–	.76 (.01)*	.01 (.01)	–.02 (.01)	.77 (.01)*
TT2G34B	–	–	.80 (.01)*	–.07 (.01)*	–.08 (.01)*	.97 (.01)*
TT2G34E	–	–	.69 (.01)*	.16 (.01)*	.07 (.01)*	.51 (.01)*
TT2G34G	–	–	.70 (.01)*	.07 (.01)*	.31 (.01)*	.39 (.01)*
*Factor Correlations*						
Factor 2	.68 (.01)*	–	–	.64 (.01)*	–	–
Factor 3	.66 (.01)*	.78 (.01)*	–	.62 (.01)*	.68 (.01)*	–
*Model Fit Indices*						
SB- χ^2^ [*df*]	4,313.7 [51]*			1,228.0 [33]*		
CFI	.950			.986		
TLI	.936			.972		
RMSEA	.023			.015		
CI_90-RMSEA_	[.022, .023]			[.014, .016]		
SRMR	.041			.014		

*Note*. Standard errors are shown in parentheses. SB- χ^2^ = Satorra-Bentler corrected χ^2^ value. CI_90-RMSEA_ = 90% confidence interval of the RMSEA, *N* = 164,687. In these analyses, the TALIS 2013 sample was considered a single-group sample.

* *p* < .01.

In the third step, we tested whether the ESEM approach was superior in each of the TALIS 2013 countries. These country-by-country analyses did not yet adopt a multi-group modeling approach and showed that–although the CFA model showed marginal to acceptable fit statistics (e.g., for Japan)–ESEM was preferred in 31 of the TALIS 2013 countries with the exception of Bulgaria (see Table 3). This was indicated by a reduction in the χ^2^ statistic, lower RMSEA values that were closer to the more restrictive cut-off of .05, lower SRMR values, and higher CFI and TLI values. As one of the prerequisites of testing these models for measurement invariance across countries is an acceptable fit of the model to the data of each country, the ESEM rather than the CFA approach can be considered a candidate for invariance testing. We note that these country-by-country analyses directly feed into the test for configural invariance, which summarizes these analyses in a multi-group model. Hence, although described under Research Question 2, the appropriate and superior fit of the configural invariance ESEM over CFA supports the preference of ESEM (please refer to the subsequent section for more details).

**Table 3:** Fit Indices and Comparisons of CFA and ESEM Models for Each Country.

*Country*	*Model*	*SB-* χ^2^ [*df*]	*CFI*	*TLI*	*RMSEA*	*CI*_*90-RMSEA*_	*SRMR*
Australia^a^	CFA	1,399.2 [51]*	.929	.908	.065	[.062, .068]	.049
	ESEM	550.0 [33]*	.973	.946	.050	[.046, .054]	.019
Brazil	CFA	844.5 [51]*	.944	.928	.034	[.032, .036]	.042
	ESEM	222.3 [33]*	.987	.973	.021	[.018, .023]	.015
Bulgaria	CFA	520.1 [51]*	.920	.897	.056	[.052, .060]	.040
	ESEM	393.8 [33]*	.939	.877	.061	[.056, .066]	.029
Chile	CFA	444.3 [51]*	.935	.916	.071	[.065, .077]	.043
	ESEM	145.3 [33]*	.982	.963	.047	[.039, .055]	.019
Croatia	CFA	838.7 [51]*	.932	.913	.065	[.061, .069]	.048
	ESEM	202.5 [33]*	.985	.971	.038	[.033, .043]	.015
Czech Republic	CFA	661.8 [51]*	.937	.918	.061	[.057, .065]	.047
	ESEM	166.3 [33]*	.986	.972	.036	[.030, .041]	.016
Denmark^b^	CFA	932.4 [51]*	.932	.912	.058	[.055, .052]	.041
	ESEM	260.8 [33]*	.982	.965	.037	[.033, .041]	.017
Estonia	CFA	703.8 [51]*	.914	.888	.065	[.061, .069]	.050
	ESEM	166.5 [33]*	.982	.965	.036	[.031, .042]	.017
Finland^b^	CFA	2,152.5 [51]*	.912	.887	.061	[.059, .063]	.056
	ESEM	592.1 [33]*	.977	.953	.039	[.036, .042]	.018
France	CFA	652.1 [51]*	.921	.898	.065	[.060, .069]	.046
	ESEM	313.9 [33]*	.963	.926	.055	[.050, .061]	.025
Israel	CFA	627.6 [51]*	.925	.903	.059	[.055, .063]	.055
	ESEM	211.4 [33]*	.977	.954	.041	[.036, .046]	.021
Italy	CFA	1,064.2 [51]*	.944	.928	.054	[.051, .057]	.045
	ESEM	367.3 [33]*	.982	.963	.038	[.035, .042]	.018
Japan^c^	CFA	576.3 [51]*	.957	.944	.055	[.051, .059]	.047
	ESEM	181.1 [33]*	.988	.976	.036	[.031, .041]	.015
Korea^c^	CFA	1,166.7 [51]*	.920	.896	.088	[.084, .092]	.050
	ESEM	366.7 [33]*	.976	.952	.060	[.054, .065]	.020
Latvia	CFA	722.5 [51]*	.912	.885	.056	[.053, .060]	.051
	ESEM	283.3 [33]*	.967	.934	.043	[.038, .047]	.021
Malaysia^c^	CFA	1,017.0 [51]*	.910	.883	.080	[.076, .084]	.060
	ESEM	211.4 [33]*	.983	.967	.043	[.037, .048]	.016
Mexico	CFA	505.8 [51]*	.960	.949	.031	[.028, .033]	.034
	ESEM	148.0 [33]*	.990	.980	.019	[.016, .022]	.013
Netherlands	CFA	288.6 [51]*	.946	.930	.051	[.045, .057]	.039
	ESEM	156.2 [33]*	.972	.944	.046	[.039, .053]	.022
Norway^b^	CFA	881.8 [51]*	.944	.927	.047	[.044, .049]	.049
	ESEM	267.5 [33]*	.984	.968	.031	[.027, .034]	.017
Poland	CFA	1,561.3 [51]*	.922	.899	.054	[.052, .056]	.048
	ESEM	398.8 [33]*	.981	.962	.033	[.030, .036]	.017
Portugal	CFA	1,336.7 [51]*	.920	.896	.061	[.059, .064]	.052
	ESEM	327.0 [33]*	.982	.963	.036	[.033, .040]	.017
Serbia	CFA	827.5 [51]*	.924	.902	.063	[.059, .067]	.048
	ESEM	311.7 [33]*	.973	.946	.047	[.042, .052]	.020
Singapore^c^	CFA	3,540.7 [51]*	.925	.903	.081	[.079, .084]	.049
	ESEM	989.8 [33]*	.979	.959	.053	[.050, .056]	.016
Slovak Republic	CFA	905.8 [51]*	.913	.887	.070	[.066, .074]	.048
	ESEM	285.8 [33]*	.974	.948	.047	[.042, .052]	.019
Spain	CFA	1,695.6 [51]*	.924	.902	.059	[.057, .061]	.056
	ESEM	402.0 [33]*	.983	.966	.035	[.032, .038]	.017
Sweden^b^	CFA	696.1 [51]*	.933	.913	.063	[.059, .067]	.055
	ESEM	214.4 [33]*	.981	.962	.042	[.036, .047]	.019
United States of	CFA	468.8 [51]*	.922	.900	.066	[.061, .072]	.064
America^a^	ESEM	186.9 [33]*	.971	.943	.050	[.043, .057]	.023
Sub-national entities							
England (United	CFA	586.1 [51]*	.922	.900	.067	[.062, .072]	.051
Kingdom) ^a^	ESEM	240.3 [33]*	.970	.940	.052	[.046, .058]	.022
Flanders (Belgium)	CFA	1,145.5 [51]*	.923	.901	.062	[.058, .065]	.053
	ESEM	303.2 [33]*	.981	.962	.038	[.034, .042]	.017
Abu Dhabi (United Arab Emirates)	CFA	639.7 [51]*	.924	.902	.050	[.047, .054]	.046
	ESEM	270.4 [33]*	.969	.939	.040	[.036, .044]	.020
Alberta (Canada)	CFA	588.7 [51]*	.923	.900	.078	[.073, .084]	.055
	ESEM	230.5 [33]*	.972	.943	.059	[.052, .066]	.021
Romania	CFA	523.4 [51]*	.921	.898	.038	[.035, .041]	.047
	ESEM	225.4 [33]*	.968	.936	.030	[.026, .034]	.022

*Note*. SB- χ^2^ = Satorra-Bentler corrected χ^2^ value. CI_90-RMSEA_ = 90% confidence interval of the RMSEA, CFA = Confirmatory Factor Analysis, ESEM = Exploratory structural equation modeling.

^a^ Anglo-Saxon country cluster

^b^ Nordic country cluster

^c^ East and South-East Asian country cluster.

* *p* < .01.

In light of these results on the baseline factor structure, we accepted the ESEM approach as a better representation of the structure of the TSES scale than the CFA approach. We therefore focused the investigation of the different levels of measurement invariance on ESEM, yet reporting the results of CFA invariance testing.

### Measurement Invariance (Research Question 2)

Our second research question was aimed at testing whether or not the factor structure of the TSES measure–as identified under Research Question 1 –could be generalized across countries. To answer this question, we first applied measurement invariance testing to the ESEM approach (see Table 4).

**Table 4:** Fit Indices and Comparisons of ESEM Invariance Models (32-countries group and country clusters).

*Model*	*SB-* χ^2^ [*df*]	*CFI*	*TLI*	*RMSEA*	*CI*_*90-RMSEA*_	*SRMR*	Δ*CFI*	Δ*TLI*	Δ*RMSEA*	Δ*SRMR*
Total TALIS 2013 Sample										
Configural invariance	9,489.4 [1,056]*	.979	.958	.039	[.039, .040]	.018	–	–	–	–
Metric invariance	15,288.1 [1,893]*	.967	.963	.037	[.037, .038]	.041	–.012	+.005	–.002	+.023
Scalar invariance	43,780.5 [2,172]*	.896	.899	.061	[.061, .062]	.071	–.071	–.064	+.024	+.030
Strict invariance	60,441.1 [2,544]*	.855	.880	.066	[.066, .067]	.121	–.041	–.019	+.005	+.050
East and South-East Asian countries (Japan, Korea, Malaysia, Singapore)										
Configural invariance	1,719.3 [132]*	.981	.961	.050	[.048, .052]	.016	–	–	–	–
Metric invariance	2,614.5 [213]*	.971	.964	.048	[.046, .050]	.036	–.010	+.003	–.002	+.020
Partial scalar invariance	3,489.4 [231]*	.960	.955	.054	[.052, .055]	.042	–.011	–.009	+.006	+.020
Scalar invariance	5,579.1 [240]*	.935	.929	.067	[.066, .069]	.052	–.025	–.016	+.006	+.010
Strict invariance	6,679.8 [276]*	.922	.925	.069	[.067, .070]	.077	–.013	–.004	+.002	+.025
Anglo-Saxon countries (Australia, England, Unites States of America)										
Configural invariance	954.6 [99]*	.972	.945	.050	[.047, .053]	.020	–	–	–	–
Metric invariance	1,052.0 [153]*	.971	.963	.041	[.039, .043]	.026	–.001	+.018	–.009	+.006
Partial scalar invariance	1,143.0 [165]*	.969	.962	.041	[.039, .043]	.029	–.002	–.001	–.009	+.006
Scalar invariance	1,261.6 [171]*	.965	.959	.043	[.041, .045]	.032	–.004	–.003	+.002	+.003
Strict invariance	1,291.5 [195]*	.965	.964	.040	[.038, .042]	.040	.000	+.005	–.003	+.008
Nordic countries (Denmark, Finland, Norway, Sweden)										
Configural invariance	1,434.4 [132]*	.980	.960	.038	[.037, .040]	.018	–	–	–	–
Metric invariance	2,016.3 [213]*	.973	.966	.036	[.034, .037]	.033	–.007	+.006	–.002	+.015
Partial scalar invariance	3,209.4 [231]*	.955	.948	.044	[.043, .045]	.039	–.018	–.018	+.008	+.006
Scalar invariance	5,307.7 [240]*	.923	.915	.056	[.055, .057]	.056	–.022	–.033	+.012	+.017
Strict invariance	6,429.5 [276]*	.907	.911	.058	[.056, .059]	.082	–.016	–.004	+.002	+.026

*Note*. SB- χ^2^ = Satorra-Bentler corrected χ^2^ value. CI_90-RMSEA_ = 90% confidence interval of the RMSEA.

* *p* < .01. For the total TALIS 2013 sample, partial scalar was not tested due to a large number of possible combinations that could be used to constrain some of the item intercepts across the 32 participating countries.

Applying the previously identified, criteria to evaluate changes in model fit but taking into account that these criteria, we evaluated the results of invariance testing as follows: For the *total TALIS 2013 sample*, the ESEM approach revealed metric invariance across the 32 participating countries, but failed to detect scalar and strict invariance. As a consequence, only comparisons of factor correlations or relations to, for instance, external constructs can be compared. We noticed that only the change in the CFI was slightly higher than the suggested cut-off (ΔCFI = –.012); yet, all other criteria were met. As noted previously, changes in fit statistics are not equally sensitive to invariance and the suggested cut-offs are not to be regarded as golden rules. In fact, small deviations from these cut-off values can still be accepted and do not indicate substantial deviation from non-invariance [9]. For the *Nordic* cluster, the *metric invariance* model represented the most acceptable model whereas assuming stricter levels of invariance lead to a substantial decrease in model fit (ΔCFI = –.018, ΔTLI = –.018). This finding implies that mean comparisons across the Nordic countries should not be employed; however, the pattern of factor correlations can be compared across countries [14]. For the *East and South-East Asian* countries, the change in the CFI only slightly exceeded the suggested cut-off for the CFI (ΔCFI = –.011); yet, all other cut-off criteria were met. We therefore accepted the partial scalar invariance model. Since only one item intercept per factor was freely estimated in this model, mean comparisons can still be employed [68, 78]. Specifically, the intercepts of items TT2G34E, TT2G34F, and TT2G34L varied. Finally, the invariance testing across the *Anglo-Saxon* countries revealed that *strict invariance* was given. Hence, for these countries, mean comparisons of the TSES factors can be employed using their factor scores or sum scores.

In a second step, we conducted the invariance analyses for the CFA approach, although the baseline models indicated a strong preference for the ESEM approach, and showed only substantial fit statistics for each of the 32 countries. The results are detailed in Table 5 and indicate changes in goodness-of-fit similar to those in ESEM. Specifically, the CFA approach led to metric invariance for the total sample, partial scalar invariance for the East and South-East Asian country cluster, strict invariance for the Anglo-Saxon country cluster, and metric invariance for the Nordic country cluster. However, as the ESEM invariance models significantly outperformed the CFA invariance models in terms of model fit for both the total TALIS 2013 sample and the selected country clusters, we regarded this as support for the preference of ESEM.

**Table 5:** Fit Indices and Comparisons of CFA Invariance Models (32-countries group and country clusters).

*Model*	*SB-* χ^2^ [*df*]	*CFI*	*TLI*	*RMSEA*	*CI*_*90-RMSEA*_	*SRMR*	Δ*CFI*	Δ*TLI*	Δ*RMSEA*	Δ*SRMR*
Total TALIS 2013 sample										
Configural invariance	30,275.3 [1,632]*	.928	.907	.058	[.058, .059]	.049	–	–	–	–
Metric invariance	33,222.1 [1,911]*	.922	.913	.056	[.056, .057]	.058	–.006	+.006	–.002	+.009
Scalar invariance	63,041.3 [2,190]*	.848	.853	.073	[.073, .074]	.082	–.074	–.060	+.017	+.024
Strict invariance	79,986.0 [2,562]*	.806	.840	.077	[.076, .077]	.130	–.042	–.013	+.004	+.047
East and South-East Asian countries (Japan, Korea, Malaysia, Singapore)										
Configural invariance	6,239.1 [204]*	.927	.905	.078	[.076, .079]	.050	–	–	–	–
Metric invariance	6,730.3 [231]*	.921	.910	.076	[.074, .077]	.056	–.006	+.005	–.002	+.006
Partial scalar invariance	7,556.9 [249]*	.911	.906	.078	[.076, .079]	.057	–.010	–.004	+.002	+.001
Scalar invariance	10,371.3 [258]*	.877	.874	.090	[.088, .091]	.070	–.034	–.032	+.012	+.017
Strict invariance	11,267.8 [294]*	.866	.880	.087	[.086, .089]	.091	–.011	–.006	–.003	+.021
Anglo-Saxon countries (Australia, England, Unites States of America)										
Configural invariance	2,421.5 [153]*	.927	.906	.065	[.063, .067]	.053	–	–	–	–
Metric invariance	2,507.7 [171]*	.925	.913	.063	[.060, .065]	.054	–.002	+.007	–.002	+.001
Partial scalar invariance	2,602.7 [183]*	.922	.916	.062	[.059, .064]	.056	–.003	+.003	–.001	+.002
Scalar invariance	2,752.2 [189]*	.918	.914	.062	[.060, .064]	.059	–.004	–.002	.000	+.003
Strict invariance	2,783.4 [213]*	.917	.923	.059	[.057, .061]	.063	–.001	+.009	–.003	+.004
Nordic countries (Denmark, Finland, Norway, Sweden)										
Configural invariance	5,041.0 [204]*	.927	.905	.059	[.058, .061]	.051	–	–	–	–
Metric invariance	5,401.5 [231]*	.922	.910	.058	[.056, .059]	.057	–.005	–.005	–.001	+.006
Partial scalar invariance	6,619.1 [249]*	.903	.897	.062	[.061, .063]	.062	–.019	–.013	+.004	+.005
Scalar invariance	9,645.8 [258]*	.857	.854	.074	[.072, .075]	.076	–.046	–.043	+.012	+.015
Strict invariance	10,905.8 [294]*	.839	.855	.073	[.072, .075]	.105	–.018	+.001	–.001	+.029

*Note*. SB- χ^2^ = Satorra-Bentler corrected χ^2^ value. CI_90-RMSEA_ = 90% confidence interval of the RMSEA. For the total TALIS 2013 sample, partial scalar was not tested due to a large number of possible combinations that could be used to constrain some of the item intercepts across the 32 participating countries.

* *p* < .01.

As a final step of comparing the CFA and ESEM approaches, we investigated the factor correlations on the basis of the metric invariance models. Table 6 details these correlations for each of the 32 countries. In general, a tendency of lower correlations in the ESEM approach could be observed, which was also apparent in the total sample data (see Table 2). This observation was due to the existence of cross-loadings in ESEM. Specifically, the factor correlations ranged between .50 and .90; the highest coefficients were obtained for the relation between teachers’ self-efficacy in instruction and student engagement. Whereas most correlations differed only slightly between CFA and ESEM, there was considerable cross-country variation in the relation between the scales “Classroom management” and “Student engagement”–the two scales that indicated a substantial overlap in many countries.

**Table 6:** Correlations among the three Factors of Teachers’ Sense of Self-Efficacy for CFA and ESEM.

*Country*	*Correlations* ρ (CFA/ESEM)		
	*Classroom Management–Instruction*	*Classroom Management–Student Engagement*	*Instruction–Student Engagement*
Australia^a^	.69*/ .64*	.60*/ .55*	.74*/ .64*
Brazil	.66*/ .61*	.75*/ .67*	.76*/ .65*
Bulgaria	.68*/ .65*	.74*/ .67*	.84*/ .75*
Chile	.79*/ .75*	.81*/ .73*	.85*/ .73*
Croatia	.67*/ .59*	.64*/ .55*	.73*/ .60*
Czech Republic	.59*/ .52*	.59*/ .53*	.74*/ .59*
Denmark^b^	.61*/ .56*	.68*/ .61*	.81*/ .68*
Estonia	.65*/ .61*	.68*/ .56*	.79*/ .63*
Finland^b^	.63*/ .58*	.63*/ .56*	.79*/ .65*
France	.59*/ .53*	.60*/ .54*	.73*/ .63*
Israel	.65*/ .60*	.65*/ .59*	.77*/ .68*
Italy	.62*/ .55*	.69*/ .64*	.79*/ .70*
Japan^c^	.68*/ .64*	.64*/ .62*	.83*/ .78*
Korea^c^	.83*/ .80*	.84*/ .77*	.90*/ .82*
Latvia	.60*/ .53*	.58*/ .51*	.64*/ .50*
Malaysia^c^	.73*/ .69*	.76*/ .71*	.87*/ .77*
Mexico	.67*/ .62*	.68*/ .63*	.82*/ .73*
Netherlands	.62*/ .57*	.64*/ .59*	.79*/ .71*
Norway^b^	.58*/ .51*	.67*/ .61*	.75*/ .60*
Poland	.69*/ .64*	.65*/ .55*	.81*/ .65*
Portugal	.64*/ .60*	.69*/ .61*	.76*/ .67*
Serbia	.65*/ .59*	.69*/ .61*	.72*/ .59*
Singapore^c^	.75*/ .71*	.74*/ .67*	.82*/ .73*
Slovak Republic	.74*/ .69*	.78*/ .67*	.83*/ .67*
Spain	.61*/ .54*	.68*/ .62*	.72*/ .60*
Sweden^b^	.56*/ .49*	.68*/ .62*	.82*/ .72*
United States of America^a^	.61*/ .55*	.57*/ .53*	.68*/ .57*
Sub-national entities			
England (United Kingdom)^a^	.67*/ .63*	.66*/ .59*	.73*/ .64*
Flanders (Belgium)	.57*/ .50*	.61*/ .54*	.78*/ .63*
Abu Dhabi (United Arab Emirates)	.71*/ .66*	.78*/ .70*	.77*/ .66*
Alberta (Canada)	.62*/ .57*	.59*/ .54*	.71*/ .61*
Romania	.75*/ .68*	.67*/ .61*	.69*/ .62*

*Note*. In each cell, the first correlation reported was obtained from the CFA and the second from the ESEM approach.

^a^ Anglo-Saxon country cluster

^b^ Nordic country cluster

^c^ East and South-East Asian country cluster.

* *p* < .01.

Taken together, within each country cluster and for the entire TALIS 2013 sample, at least metric invariance could be established using ESEM, showing that the TSES factor structure (i.e., number of factors and factor loadings) is robust. Furthermore, for the East and South-East Asian as well as the Anglo-Saxon countries mean comparisons were meaningful. Please find the corresponding mean comparisons in the S2 Table. We noticed that the use of ESEM improved the invariance within the selected country clusters but not across the entire set of 32 participating countries.

### Correlations with External Constructs (Research Question 3)

To address our third research question, we investigated the correlations among teachers’ self-efficacy, their years of work experience and job satisfaction with the teaching profession. Given that metric invariance was met for both the CFA and ESEM approach across all TALIS 2013 countries, it was possible to compare the relations to these external constructs by using multi-group CFA and ESEM models of metric invariance. Table 7 shows the resulting correlations.

**Table 7:** Correlations among the three Factors of the TSES measure and External Constructs (Years of Work experience and Job Satisfaction) for CFA and ESEM.

*Sample*	*Correlations* ρ *(CFA/ESEM)*					
	*Classroom Management–Work experience*	*Instruction–Work experience*	*Student Engagement–Work experience*	*Classroom Management–Job Satisfaction*	*Instruction–Job Satisfaction*	*Student Engagement–Job Satisfaction*
Australia^a^	.11*/ .10*	.13*/ .12*	.11*/ .11*	.23*/ .22*	.23*/ .22*	.26*/ .25*
Brazil	.10*/ .11*	-.01/ -.01	.08*/ .07*	.19*/ .19*	.23*/ .22*	.30*/ .28*
Bulgaria	-.05/ -.05	-.10*/ -.09*	-.05/ -.06	.26*/ .27*	.19*/ .17*	.27*/ .26*
Chile	.10*/ .11*	.02/ .01	.05/ .05	.25*/ .24*	.24*/ .23*	.32*/ .32*
Croatia	.16*/ .16*	.20*/ .18*	.25*/ .24*	.32*/ .31*	.27*/ .25*	.37*/ .35*
Czech Republic	.14*/ .14*	.06/ .03	.06/ .06	.21*/ .21*	.19*/ .17*	.23*/ .22*
Denmark^b^	.18*/ .18*	.13*/ .11*	.14*/ .14*	.29*/ .29*	.27*/ .25*	.28*/ .27*
Estonia	.05/ .06	.03/ -.03	.03/ .02	.10*/ .09*	.19*/ .17*	.28*/ .27*
Finland^b^	.07*/ .08*	.00/ .00	.06*/ .05*	.25*/ .25*	.29*/ .27*	.33*/ .31*
France	.16*/ .16*	.09*/ .07*	.18*/ .18*	.22*/ .22*	.20*/ .19*	.21*/ .20*
Israel	.07*/ .07*	.05/ .05	.07*/ .07*	.29*/ .29*	.20*/ .19*	.28*/ .27*
Italy	.21*/ .21*	.06*/ .05*	.09*/ .08*	.20*/ .20*	.21*/ .19*	.26*/ .25*
Japan^c^	.11*/ .11*	.12*/ .11*	.21*/ .21*	.27*/ .26*	.23*/ .22*	.25*/ .25*
Korea^c^	.10*/ .10*	.07*/ .06*	.07*/ .06*	.28*/ .29*	.26*/ .26*	.31*/ .28*
Latvia	.11*/ .11*	.05/ .04	.10*/ .09*	.16*/ .16*	.21*/ .19*	.28*/ .28*
Malaysia^c^	.03/ .03	.03/ .03	.10*/ .10*	.33*/ .32*	.37*/ .35*	.41*/ .40*
Mexico	.06*/ .07*	.01/ .01	.04/ .03	.25*/ .24*	.34*/ .33*	.37*/ .36*
Netherlands	.09*/ .08*	.03/ .03	.11*/ .12*	.27*/ .27*	.26*/ .24*	.32*/ .31*
Norway^b^	.12*/ .12*	-.02/ -.04	.04/ .04	.24*/ .24*	.24*/ .21*	.27*/ .26*
Poland	.13*/ .12*	.08/ .08*	.11*/ .11*	.24*/ .23*	.25*/ .23*	.30*/ .29*
Portugal	.01/ .01	-.02/ -.02	-.01/ -.02	.25*/ .25*	.22*/ .21*	.25*/ .23*
Serbia	.08*/ .08*	.02/ .01	.13*/ .13*	.32*/ .31*	.27*/ .23*	.38*/ .37*
Singapore^c^	.16*/ .15*	.19*/ .18*	.23*/ .23*	.19*/ .18*	.20*/ .19*	.27*/ .27*
Slovak Republic	.13*/ .13*	.12*/ .11*	.15*/ .14*	.23*/ .23*	.21*/ .19*	.26*/ .25*
Spain	.06*/ .06*	-.05*/ -.06*	-.02/ -.01	.31*/ .30*	.29*/ .27*	.33*/ .32*
Sweden^b^	.15*/ .15*	.05/ .03	.13*/ .14*	.14*/ .15*	.24*/ .24*	.28*/ .25*
United States of America^a^	.14*/ .13*	.07/ .06	.05/ .05	.17*/ .16*	.16*/ .14*	.30*/ .30*
Sub-national entities						
England (United Kingdom)^a^	.09*/ .08*	.04/ .03	.10*/ .11*	.20*/ .20*	.19*/ .17*	.30*/ .29*
Flanders (Belgium)	.12*/ .12*	.08*/ .08*	.16*/ .15*	.16*/ .16*	.12*/ .09*	.13*/ .13*
Abu Dhabi (United Arab Emirates)	.17*/ .17*	.16*/ .16*	.17*/ .16*	.21*/ .20*	.23*/ .22*	.29*/ .29*
Alberta (Canada)	.18*/ .17*	.09*/ .08	.16*/ .17*	.18*/ .18*	.18*/ .16*	.25*/ .25*
Romania	.12*/ .12*	.11*/ .10*	.13*/ .12*	.23*/ .23*	.27*/ .25*	.32*/ .32*
Total TALIS 2013 Sample						
32 countries	.09*/ .09*	.03*/ .02	.04*/ .04*	.19*/ .19*	.23*/ .22*	.68*/ .64*

*Note*. In each cell, the first correlation reported was obtained from the CFA and the second from the ESEM approach.

^a^ Anglo-Saxon country cluster

^b^ Nordic country cluster

^c^ East and South-East Asian country cluster.

* *p* < .01.

Examining the correlations between the TSES factors and work experience, the total TALIS 2013 sample revealed small but significantly positive relations for all factors, suggesting that the more experience teachers have, the higher their self-efficacy. This finding was by and large replicated for the countries within the three selected clusters; nevertheless, in some countries, the correlations were insignificant. Comparing these results across the two modeling approaches (i.e., CFA versus ESEM) indicated only small differences in the correlations; in fact, they were statistically insignificant (results of the corresponding significance tests are not shown in the table for reasons of comprehensibility).

The correlations between the TSES factors and teachers’ job satisfaction with their profession were significantly positive for the total sample and each of the 32 TALIS 2013 countries, suggesting that high levels of self-efficacy were associated with high levels of job satisfaction. As for the relations to work experience, the differences in the correlations between the CFA and ESEM approach were insignificant. Hence, for both external variables, the metric invariance models of the two modeling approaches corresponded.

## Discussion

Recent research on teachers’ self-efficacy has been concerned with the appropriate measurement of these self-beliefs with a particular emphasis on its dimensionality and invariance across countries [7, 12]. Some studies found considerable evidence for the distinction between three or more facets of the TSES measure and the invariance of the underlying models across countries [10, 11]. Furthermore, researchers have pointed out that comparing self-efficacy across countries on the basis of representative large-scale assessment data is often compromised [9, 15]. Against this background, the present investigation was concerned with the modeling of teachers’ self-efficacy as a multidimensional construct by using representative TALIS 2013 data. Using exploratory structural equation modeling, we found support for our theoretical assumptions on the existence of significant cross-loadings in the factor structure of the TSES measure and obtained evidence on at least metric invariance across the 32 participating countries, and (partial) scalar invariance for some country clusters. We discuss our results in light of the potential advantages of the ESEM on the one hand, and with respect to the validity of the TSES measure on the other hand.

## Structure of the Teachers’ Sense of Self-efficacy Measure (Research Question 1)

Our first research question addressed the structure of the TSES measure and tested the assumption of perfect item-factor links, as manifested by significant item cross-loadings. The results suggested that an exploratory structural equation model with three correlated factors fitted the data significantly better than a CFA model without cross-loadings. This finding has a number of implications: First, given the acceptable model fit for the total sample and all of the country samples, we have support for the distinction between three facets of teachers’ self-efficacy, namely self-efficacy in classroom management, instruction, and student engagement. This result is in line with a number of studies who have examined the dimensionality of the TSES measure, and supports the argumentation that the construct is multifaceted [6–8, 24, 27]. Besides this conceptual perspective on the dimensionality, we can also interpret our finding as evidence for the factorial validity the self-efficacy assessment used in TALIS 2013, since there is a remarkable fit between the hypothesized and empirical structure of the construct [79]. Given that this finding did not only hold for the total sample but also for each country, the robustness of the structure and conceptualization is indicated [7]. Moreover, in line with Klassen et al. [11], the high reliabilities of the three self-efficacy factors show the accuracy of the measurement, thus meeting the prerequisites of studying construct validity. From a practitioner’s point of view and based on the multidimensional information on self-efficacy in the measurement, the needs for professional and personal development can be identified [25]. This information may be used for specific interventions on strengthening teachers’ self-efficacy and thereby enhancing their well-being and job satisfaction in order to prevent burnout and emotional exhaustion [5, 7].

Second, the differentiation of self-efficacy into three factors also shows that the construct corresponds to aspects of teaching quality such as classroom management, instructional strategies of cognitive activation, and student engagement that are at the center of research on instructional quality and often assessed at the student level [18, 19, 36, 39]. Our secondary data analyses supported this correspondence in light of the factor structure. Furthermore, this argumentation builds upon the idea that these perceptions should be assessed by items that cover a wide range of teaching practices, fit the classroom context and the requirements of teaching [25, 34]. However, further research should aim at linking the self-efficacy factors with observed or rated teaching quality factors directly [80]. Some studies have already shown a positive link between the two concepts of self-efficacy and teaching quality with respect to classroom management [3, 35].

Third, in light of the moderate to high correlations among the teacher self-efficacy factors obtained from ESEM, we furthermore argue that these results confirm previous findings that the three factors are related, indicating that teachers are generally able to distinguish between the three factors when evaluating their instructional capabilities [25, 81]. Nevertheless, a higher-order factor model comprising a second-order and three first-order factors of the TSES measure may also appear reasonable, particularly because some research suggests that for teachers with little work experience, the TSES can be better described by a single-factor model [27]. We therefore encourage further research on the potential changes in the factor structure of TSES across different levels of work experience [28].

Fourth, the distinction between the three factors is not perfect, since significant cross-loadings existed (see Table 2). Freeing the assumption of perfect item-factor links led to well-fitting measurement models. From a conceptual perspective, this result supports our expectations on the existence of a construct overlap that reflects the commonalities between the measurements of the three self-efficacy factors. Referring to research on teaching practices and quality, we argue that the concepts of classroom management, instruction, and student engagement are not clearly distinct [18]. For instance, cognitively activating activities may go together with engaging students for learning [82]. In the same way, teachers’ self-efficacy beliefs may overlap. From a measurement point of view, this overlap can also be interpreted as an item characteristic. Nevertheless, although eliminating items with cross-loadings is a common practice in the development of ‘psychometrically pure’ scales, we argue that deleting these items would compromise the conceptual breadth of the TSES measure. With the advancement of new methodologies such as ESEM, construct overlaps can be explicitly modeled in order to best represent the theoretical conception of constructs. Another possibility of coping with items that show significant cross-loadings is to revise them by changing their wording such that it becomes clearer to which factor they belong. This approach, however, requires multiple steps of test validation, as the revised items need to be evaluated repeatedly.

Taken together, we answer Research Question 1 as follows: For the total sample and the selected subsamples of countries who participated in the TALIS 2013 study, the assumption of perfect item-factor links does not hold. In our study, ESEM provides a flexible modeling approach which represents the substantive assumption on the factor structure better than CFA without cross-loadings in terms of absolute goodness-of-fit.

### Invariance of Teachers’ Self-efficacy (Research Question 2)

Our second research question was concerned with the invariance of the previously identified TSES measurement model across countries. In general, across all countries, the numbers of factors and loadings remained comparable, indicating that the factor structure is robust (see [12] for comparable findings). To some extent, this finding lends evidence on the generalizability of the three-factor structure and suggests construct validity [11]. Given that metric invariance was achieved across all participating TALIS 2013 countries, comparisons of relations to external constructs [given that they also provide at least metric invariance] can be conducted. This finding was to some extent expected, as the countries represent culturally and educationally diverse systems, in which self-beliefs may be understood differently [15]. Nevertheless, this finding is consistent with existing studies on the cross-cultural invariance of the TSES measure that used multi-group CFA [10–12, 15, 33, 49]. Even though ESEM provided a significantly better representation of TSES at the country level (see Research Question 1) and provided a modeling approach that is flexible enough to be extended to multi-group ESEM, the existence of cross-loadings did not lead to an improvement in the overall level of invariance across all countries. This finding is not surprising, because the item intercept structure is not necessarily aligned when allowing for item cross-loadings in data sets with a relatively large number of groups [43, 44].

With respect to the country clusters, the results of invariance testing suggested that different levels of invariance were given for each cluster; the ESEM factor structure is not fully invariant, because strict invariance has only been met for the cluster of Anglo-Saxon countries. In particular, the Nordic cluster showed metric invariance across countries, indicating the comparability of the number of factors and the item-factor links. Since higher levels of invariance such as scalar invariance which assume equal item intercepts did not hold, factor mean comparisons are compromised because the three factors have different meanings across countries [14]. This indication of differential item functioning might be due to different response styles of teachers in the Nordic countries, which manifest in unequal intercepts. In fact, Vieluf and colleagues [15] found support for this argument for at least Norwegian and Danish teachers who participated in TALIS 2008 and showed significant differences in their general self-efficacy. Moreover, the general response tendencies for the Nordic countries in TALIS 2013 indicated higher self-efficacy beliefs for Danish and Swedish teachers at the item level ([19], pp. 407–408). But this finding warrants further research on the particular reasoning of why the self-efficacy assessment in TALIS 2013 worked out differently in these countries. Potential sources of item bias may be related to differences in teacher education, teaching requirements, language differences, and cultural differences in teaching beliefs [51]. For the East and South-East Asian countries, partial scalar invariance with three freely estimated intercepts could be established. Hence, the comparability of factor scores is partly given. The three items that showed differential functioning referred to teachers’ self-efficacy in motivating students who show low interest in learning (TT2G34E), making expectations about student behavior clear (TT2G34F), and implementing alternative instructional strategies (TT2G34L). These items might be “culturally sensitive” or biased, since the classroom practices they refer to may vary across the Asian countries [51]. Moreover, in light of the different educational cultures in these countries [11, 19], different beliefs about teaching practices may interact with the understanding of the self-efficacy items. Finally, full invariance could be established for the Anglo-Saxon countries, lending evidence on full generalizability of the self-efficacy measure. We suspect that the language similarities and similarities in teaching practices may explain this finding [19]. Moreover, for the majority of students in these countries, the original version of the TALIS 2013 questionnaire was used, for which any translation into another language was not necessary. Hence, since the same, untranslated TSES scale was administered, translation bias did not affect the measurement.

In summary, in line most studies on cross-country comparisons that took a multidimensional perspective of teachers’ self-efficacy and only found metric or invariance of covariance matrices across educationally diverse countries such as Australia and China [10], Canada, Cyprus, Korea, Singapore, and the United States [11], and Germany and New Zealand [12], the ESEM approach, as applied in our study, led to the same result across the 32 participating TALIS 2013 countries. Nevertheless, higher levels of invariance and comparability could be achieved for two country clusters comprising educationally more similar countries. Consequently, we argue that too strict assumptions on the factor structure may cause non-invariance. Interestingly, our study shows that differences in item functioning between countries of similar cultures exist [7, 11], although it would have been more likely to find such differences for inherently different cultures [10]. We note that although our selection of country clusters was theory-driven, further statistical criteria could be developed in order to identify clusters for which scalar or even full measurement invariance may hold.

### Relations to Teachers’ Work experience and Job Satisfaction (Research Question 3)

In order to study the impact of the existence of construct overlaps as manifested by significant cross-loadings, we studied the relations among the TSES factors, teachers’ work experience, and their job satisfaction with their profession. Supporting prior research, there was a positive relation between TSES and job satisfaction for the total TALIS 2013 sample and the selected countries [1, 10, 11], whereas the positive TSES—work experience relation indicated some country-specific variation; yet, suggesting a positive association that confirms prior research [26]. Hence, the general findings on the correlations between TSES and external variables were in line with what can be expected from existing research. Interestingly, the results did not differ between the CFA and ESEM approaches, pointing to limited effects of construct overlaps on the correlations. Nevertheless, it needs to be further clarified to what extent further parameters in the TSES measurement model may explain and influence these results.

### Limitations and Future Directions

One limitation of the present investigation lies in the relatively low number and content coverage of the items which were used to measure teachers’ self-efficacy. An increased number of items representing even further facets of teachers’ self-efficacy such as the capabilities for cooperating with parents and colleagues or adapting education to individual students’ needs [6, 25] would be desirable in order to sustain construct breadth. Furthermore, the present study only focused on one TSES measure that described teachers’ general self-beliefs in their instructional capabilities; alternative measures that describe more domain-specific self-beliefs may be examined in future research. In addition, the TALIS 2013 study did not incorporate other countries from the selected clusters. For instance, besides the East and South-East Asian countries used in the present investigation, studying teachers’ self-efficacy in countries such as China or Taiwan would enhance our knowledge about cross-country differences within the cluster, especially because these countries show remarkable differences in teacher education, student achievement, and teaching practices [83, 84]. Finally, the different aspects of teachers’ self-efficacy may be related to their actual performance in classrooms [3, 4, 21]. While taking a cross-country perspective, linking teachers’ perceived instructional capabilities with their professional competence may provide meaningful insights into the determinants of instructional quality and student achievement [1, 22, 85].

### Contribution of the Study

The present study contributes to research on teachers’ self-efficacy in several ways: First, our results support the assumption of the multidimensionality of the TSES measure with three distinct but overlapping facets, and thus point to the demand of using assessments that are aligned with instructional practices [25]. Second, by using international large-scale data with representative samples and by taking a multidimensional perspective of teachers’ self-efficacy, we address the methodological challenge of invariance testing for self-efficacy measures [9, 15]. We were able to show that multi-group ESEM provides a promising approach to overcome this challenge to some extent. Third, our research responds to Malinen et al.’s [33] call for comparative studies on self-efficacy. We provide further insights into the cross-cultural generalizability of the factor structure across countries and the levels of measurement invariance attained. In this context, we regard this research as a step of construct validation with respect to the comparability and cross-national adequacy of the TSES measure and, thus, as having provided important information for researchers planning further cross-country comparisons of teachers’ self-efficacy [11, 24].

Finally, the main contribution of this research lies in the demonstration that: (a) previous findings on the distinction between three factors of teachers’ self-efficacy hold even when studying large-scale teacher samples; and (b) ESEM provides an effective tool to study cross-country measurement invariance of the TSES scale, which measures a construct that is comprised of three factors that are not strictly orthogonal. Regarding the latter contribution, ESEM may generally be a better representation of constructs with an overlapping and correlated internal structure [44].

## Conclusions

Our approach in modeling the structure and invariance of the teachers’ sense of self-efficacy measure indicated the existence of an overlap between the three factors of the construct, reflecting commonalities between the measurements of classroom management, student engagement, and instruction. Given that modeling this overlap existed in each country sample and resulted in improvements of comparability across countries of selected clusters, we hope to encourage researchers that are working on teachers’ self-efficacy to account for the overlap and thereby improve the quality of the measurement models. In this regard, we argue that for teachers’ self-efficacy, the assumption of perfect item-factor links may not fully reflect the nature of the construct. As a conclusion, we encourage researchers to consider using ESEM as an alternative and flexible approach to represent the construct in cross-cultural studies [16, 44].

Although our study also implies that researchers need to be cautious in using the TSES measurement for cross-country mean comparisons, because scalar invariance may not be achieved for larger numbers of countries, it points to the cross-cultural generalizability of the model that assumes three correlated factors of self-efficacy and imperfect item-factor links. Hence, we hope to stimulate substantive-methodological synergisms that uncover potential reasons for these cross-cultural differences and similarities on the one hand, and develop latent variable models that deal with threats of measurement invariance on the other hand. Such synergisms may bring us further in our quest for comparability.

## Supporting Information

S1 Table Items Measuring Teachers’ Self-Efficacy (OECD, 2014b, p. 195). *Note*. The item labels represent those used in TALIS 2013.
(DOCX)

S2 Table Factor Means of Teachers’ Self-Efficacy across Countries. *Note*. # Reference country. Standard deviations of factors were standardized to 1. * *p* < .01.
(DOCX)

S3 Table General information about the TALIS Board of Participating Countries (BPC). (DOCX)
