# Supplementary material for: The Quest for Comparability: Studying the Invariance of the Teachers’ Sense of Self-Efficacy (TSES) Measure across Countries
Source: PLoS One. 2016 Mar 9;11(3):e0150829. doi: 10.1371/journal.pone.0150829 (PMC4784889; doi:10.1371/journal.pone.0150829)
Supplement: S1 Table — Note. The item labels represent those used in TALIS 2013. (DOCX) [file pone.0150829.s001.docx]

# Supporting Information S1

**Table S1. Items Measuring Teachers’ Self-Efficacy ([62], p. 195).**

| *Item Wordings* | *Item Label* |
| --- | --- |
| *In your teaching, to what extent can you do the following?*  *(1 = not at all, 2 = to some extent, 3 = quite a bit, 4 = a lot)* |  |
| **Self-Efficacy in Classroom Management** |  |
| Control disruptive behaviour in the classroom | TT2G34D |
| Make my expectations about student behaviour clear | TT2G34F |
| Get students to follow classroom rules | TT2G34H |
| Calm a student who is disruptive or noisy | TT2G34I |
| **Self-Efficacy in Instruction** |  |
| Craft good questions for my students | TT2G34C |
| Use a variety of assessment strategies | TT2G34J |
| Provide an alternative explanation for example when students are confused | TT2G34K |
| Implement alternative instructional strategies in my classroom | TT2G34L |
| **Self-Efficacy in Student Engagement** |  |
| Get students to believe they can do well in school work | TT2G34A |
| Help my students value learning | TT2G34B |
| Motivate students who show low interest in school work | TT2G34E |
| Help students think critically | TT2G34G |

*Note.* The item labels represent those used in TALIS 2013.
