# Supplementary material for: The Quest for Comparability: Studying the Invariance of the Teachers’ Sense of Self-Efficacy (TSES) Measure across Countries
Source: PLoS One. 2016 Mar 9;11(3):e0150829. doi: 10.1371/journal.pone.0150829 (PMC4784889; doi:10.1371/journal.pone.0150829)
Supplement: S2 Table — Note. # Reference country. Standard deviations of factors were standardized to 1. * p < .01. (DOCX) [file pone.0150829.s002.docx]

# Supporting Information S2

**Mean Differences in Latent Variables across Countries within the Selected Country Clusters**

**Table S2. Factor Means of Teachers’ Self-Efficacy across Countries.**

| *Country* | *Factor Means in Self-Efficacy* | | |
| --- | --- | --- | --- |
|  | *Classroom Management* | *Instruction* | *Student Engagement* |
| *East and South-East Asian Countries* |  |  |  |
| Japan^#^ | 0.00 | 0.00 | 0.00 |
| Korea | 0.66* | 0.95* | 1.38* |
| Malaysia | 2.22* | 1.88* | 2.81* |
| Singapore | 0.92* | 1.31* | 1.72* |
|  |  |  |  |
| Korea^#^ | 0.00 | 0.00 | 0.00 |
| Malaysia | 1.37* | 0.87* | 1.13* |
| Singapore | 0.29* | 0.40* | 0.45* |
|  |  |  |  |
| Malaysia^#^ | 0.00 | 0.00 | 0.00 |
| Singapore | –0.74* | –0.39* | –0.42* |
|  |  |  |  |
| *Anglo-Saxon Countries* | | | |
| Australia^#^ | 0.00 | 0.00 | 0.00 |
| United States of America | 0.00 | 0.00 | –0.08 |
| England (United Kingdom) | 0.21* | 0.10* | 0.27* |
|  |  |  |  |
| United States of America^#^ | 0.00 | 0.00 | 0.00 |
| England (United Kingdom) | 0.21* | 0.10 | 0.37* |

*Note.* ^#^Reference country. Standard deviations of factors were standardized to 1. * *p* < .01.

**Description of results.** On the basis of the partial scalar invariance ESEM model, we compared the means of teachers’ self-efficacy within the East and South-East Asian cluster. Using Japan as a reference, Korean, Malaysian, and Singaporean teachers reported significantly higher self-efficacies; Malaysian teachers showed the highest factor means in all three factors. We subsequently varied the reference country to test for the significance of further mean differences. The results indicated that even when comparing Singaporean or Korean with Malaysian teachers, the latter showed significantly higher means in self-efficacy. These tendencies were supported by the mean tendencies of item responses for these countries (see [19], pp. 407–408). It is still to be examined to what extent these tendencies represent actual response styles or biases. For now, we do see evidence that teachers in Malaysia had more confidence in their teaching capabilities than teachers from the other Asian countries.

Using the strict invariance ESEM model for the Anglo-Saxon countries, we found the highest factor means for teachers in England, whereas Australia and the USA showed relatively low means. Furthermore, teachers in England reported significantly higher self-efficacies in classroom management and student engagement, yet not in instruction. Again, these tendencies mirror those found at the item level (see [19], pp. 407–408).

In total, the ESEM approach provided evidence that mean differences across countries within the East and South-East Asian and the Anglo-Saxon countries existed.

**Discussion of factor mean differences.** Given the outcomes of mean comparisons, the effect sizes were rather small for the Anglo-Saxon but high for the Asian countries. This finding suggests that teachers’ beliefs in their instructional capabilities differ only slightly within the first cluster but substantially within the second cluster. Klassen et al. [7] claimed that, even across collectivist Asian cultures, teachers’ self-efficacy may vary considerably due to differences in teacher education, in-group expectations, and demands from students. Again, potential explanations for these findings may be linked to how teaching practice is organized in these countries and how cultural values affect teachers’ beliefs [11, 19, 50-51]. In line with van de Vijver [13], we believe that further research is necessary to study the causes and consequences of these differences from cultural and educational perspectives. Nevertheless, the ESEM approach allowed us to describe these differences.
