# Supplementary material for: The Quest for Comparability: Studying the Invariance of the Teachers’ Sense of Self-Efficacy (TSES) Measure across Countries
Source: PLoS One. 2016 Mar 9;11(3):e0150829. doi: 10.1371/journal.pone.0150829 (PMC4784889; doi:10.1371/journal.pone.0150829)
Supplement: S3 Table — (DOCX) [file pone.0150829.s003.docx]

# Supporting Information S3

**General information about the TALIS Board of Participating Countries (BPC)**

The “TALIS Board of Participating Countries (BPC)” is comprised of representatives of all countries participating in TALIS. Representatives are appointed by their education ministries. Guided by the OECD’s education objectives, the Board determines the policy priorities for TALIS and makes sure that these are respected during the implementation of each TALIS survey, particularly with respect to research ethics. Prior to the main survey, the BPC approved the standards concerning survey ethics, confidentiality, and survey operations for this study. The BPC was chaired by Anne-Berit Kavli.

On approval of the BPC, the OECD released public-use data files, which can be used by researchers in order to conduct secondary data analysis. “To protect the confidentiality of the respondents, certain disclosure avoidance measures were applied at the international level which are consistent for all countries, and also at the national level which concern only specific national datasets.” (TALIS User Guide for the International Database, p. 23)

Hence, since the ethics standards for the TALIS 2013 study have been approved by the committee “TALIS Board of Participating Countries (BPC)”, further approval for the present study, which represents a secondary data analysis, is not necessary, because the TALIS 2013 international data are available publicly for research purposes.

**Members of the TALIS Board of Participating Countries for TALIS 2013**

**Australia:** Paul Hunt; Margaret Pearce; Mark Unwin **Abu Dhabi (United Arab Emirates)**: Masood Badri; Rabih Abouchakra; Tarek El Mourad; Hussein Al-Hindawi **Alberta (Canada)**: Marie-France Chouinard; Greg Rudolf; Janusz Zieminski **Brazil**: Daniel Jaime Capistrano de Olivera; Ana Carolina Silva Cirotto; Juliana Marquez Da Silva **Bulgaria**: Neda Oscar Kristanova; Marina Mavrodieva **Chile**: Violeta Aranciba Clavel; Carolina Velasco Ortúzar **Croatia**: Michelle Bras Roth **Czech Republic**: Jana Pale ková; Lubomír Martinec **Denmark**: Elsebeth Aller **England (United Kingdom)**: Lorna Bertrand **Estonia**: Priit Laanoja **Finland**: Kimmo Hämäläinen **Flanders (Belgium)**: Isabelle Erauw **France**: Jean-François Chesné; Catherine Moisan; Florence Lefresne; Caroline Simonis-Sueur **Iceland**: Julius Björnsson **Israel**: Hany Shilton; Hagit Glickman **Italy**: Maria Gemma de Sanctis; Antonella Tozza **Japan**: Tsutomu Takaguchi; Akiko Ono; Kenichi Fujioka **Korea**: Miran Jang; Doki Kim; Kapsung Kim **Latvia**: Ennata Kivrina **Malaysia**: Norlida Ab Wahab; Faizulizami Osmin **Mexico**: Ana Maria Aceves Estrada; Marina Jazmin Santos Insua **Netherlands**: Hans Ruesink **Norway**: Anne-Berit Kavli **Poland**: Lidia Olak; Magdalena Krawczyk-Radwan; Kamila Hernik **Portugal**: Nuno Rodrigues **Romania**: Silviu Cristian Mirescu **Serbia**: Danijela Petrovic **Singapore**: Siew Hoong Wong **Slovak Republic**: Romana Kanovska **Spain**: Carmen Tovar Sanchez; Javier Munoz Sanchez- Brunete; José Antonio Blanco Fernandez **Sweden**: Katalin Bellaagh **United States**: Patrick Gonzales.
